# Effects of Message Framing on Human Papillomavirus Vaccination: Systematic Review

**DOI:** 10.2196/52738

**Published:** 2024-11-07

**Authors:** Jie Gong, Dandan Gu, Suyun Dong, Wangqin Shen, Haiou Yan, Juan Xie

**Affiliations:** 1 Department of Nursing Affiliated Hospital of Nantong University Nursing and Rehabilitation School of Nantong University Nantong China; 2 School of Nursing and Rehabilitation Nantong University Nantong China

**Keywords:** message framing, gain-loss framing, human papillomavirus, vaccination, attitude, intention, behavior, systematic review, PRISMA

## Abstract

**Background:**

With the advancement of cervical cancer elimination strategies, promoting human papillomavirus (HPV) vaccination is essential to achieving this goal. The issue of how to structure and develop message content to promote HPV vaccination is a debatable issue.

**Objective:**

The efficacy of gain-loss framing in vaccination contexts is disputed. Our study aimed to elucidate the consequences of message framing on attitudes, intentions, and behavioral tendencies toward HPV vaccination, with the objective of refining message framing strategies and their elements.

**Methods:**

This systematic review adhered strictly to the PRISMA (Preferred Reporting Items for Systematic Reviews and Meta-Analysis) guideline reporting standards to comprehensively retrieve, extract, and integrate data. We searched databases, including PubMed, Embase, Scopus, and Web of Science, for literature published from database construction to August 15, 2023. Literature screening, data extraction, and quality evaluation were performed by 2 researchers. Intervention studies published in English, conducted with populations with children eligible for HPV vaccination, and involving message framing were included. Attitudes, intentions, and behaviors served as outcome evaluation criteria.

**Results:**

A total of 19 intervention studies were included. Gain-loss framing had no clear effect on vaccination attitudes nor intentions. Loss framing showed a weak advantage at improving HPV vaccination attitudes or intentions, but the evidence was not strong enough to draw definitive conclusions. The impact of gain-loss framing on HPV vaccination behaviors could not be determined due to the limited number of studies and the qualitative nature of the analysis.

**Conclusions:**

Combining gain-loss framing with other message framing approaches may be an effective way to enhance the effect of message framing. More high-quality message framing content and exploring alternative moderator or mediator variables are required to support the conclusion.

**Trial Registration:**

CRD42023451612; https://www.crd.york.ac.uk/PROSPERO/display_record.php?RecordID=451612

## Introduction

Almost 100% of cervical cancers are associated with persistent high-risk human papillomavirus (HPV) infection [1], and HPV 16 and 18, the 2 oncogenic genotypes, are the cause of more than 70% of all cervical cancers around the world [2]. In addition, 90% of warts, 88% of anal cancers, 50% of penile cancers, and 43% of vulvar cancers are attributed to HPV infection [3]. HPV vaccination prevents more than 90% of cancers and precancerous lesions caused by HPV and minimizes the morbidity or mortality associated with HPV-related diseases, while the incidence of adverse events arising from HPV vaccination is relatively modest [4]. The rate of HPV vaccination is reportedly 15% worldwide and is significantly different across nations: 71.5% in the United States, 89.5% in Britain, 8.8% in Singapore, and 2.4%-9.1% in Hong Kong, China [5,6]. Therefore, increasing the rate of HPV vaccination to prevent and control infectious diseases caused by HPV is a global issue that is worth paying attention to.

“Message framing” refers to the distinctive “framing effects” of messages [7]. The essence of “framing” is to select and emphasize messages, and the specific structure and emphasis of messages create different categories of message framing that allow people to view problems through a variety of perspectives and ultimately influence behavioral preferences [8,9]. The most classic type of framing is gain-loss framing, with the gain framing emphasizing the positive outcomes that will result in someone taking action (or negative outcomes that will be avoided) and loss framing emphasizing the negative consequences that will result in not doing something (or positive consequences that will be lost) [10-13]. Message framing plays an essential role in the dissemination of health information and persuading recipients to make behavioral modifications, with the core purpose being to orient them to modify attitudes, intentions, or behaviors toward specific health hot spots [14,15]. According to the theory of reasoned action, attitudes and intentions predict behavior, while attitudes, intentions, and behaviors measure the effectiveness of health messages [16-18].

Controversial effects of gain-loss framing on disease prevention behaviors related to vaccination have been observed. Previous reviews have shown that gain framing was more effective at persuasion for disease prevention scenarios, while loss framing worked better in terms of disease detection [19]. However, Lee and Aaker [20] proposed, based on 6 experiments, that gain framing is more appealing when the message content and settings focus on promoting and facilitating certain behaviors, whereas loss framing is more effective at preventing a phenomenon to occur. However, O'Keefe and Nan [21] performed a meta-analysis to address the influences of message framing on vaccination that revealed no difference between gain and loss framing. Therefore, the aim of our study was to systematically review the effects of interventions based on gain-loss framing for attitudes, intentions, or behaviors related to HPV vaccination and to provide directions and recommendations for designing effective message content.

## Methods

### Search Strategy

This systematic review is reported following the PRISMA (Preferred Reporting Items for Systematic Reviews and Meta-Analyses) guidelines [22]. The study protocol was registered in PROSPERO (number CRD42023451612). According to the registered protocol, we initially planned to use RevMan 5.4 software for data synthesis and meta-analysis. However, due to the heterogeneity of the included studies and the limited availability of comparable data, we were unable to perform a quantitative analysis. Therefore, we conducted a qualitative analysis to synthesize the findings. This deviation from the protocol is reported here to ensure transparency.

Computerized retrieval was conducted across 4 databases—PubMed, Embase, Scopus, and Web of Science—with the search period spanning from the inception of each database to August 15, 2023. A combination of MESH terms and keywords was adopted and adjusted to the respective features of the databases. We included 3 essential components in the search strategy and connected them with “AND”: (1) ‘message framing’ or ‘message fram*’ or ‘information framing’ or ‘information framework’ or ‘framing effect*’ or ‘gain fram*’ or ‘loss fram*’ or ‘positive fram*’ or ‘negative fram*’; (2) ‘Human papillomavirus viruses’ or ‘human papillomavirus virus’ or ‘human papillomavirus’ or ‘HPV human papillomavirus’; (3) ‘vaccination’ or vaccin*’ or ‘active immunization’ or ‘mass vaccination’ or ‘vaccination refusal’ or ‘anti-vaccination movement.’ In addition, the references of included studies were searched to obtain supplementary materials.

### Study Selection

The recommended age for HPV vaccination is between 9 years and 14 years for both boys and girls, and the decision to administer the vaccination in cases involving minors is typically made by their legal guardians [23,24]. The inclusion criteria consisted of studies that (1) included participants and any of their children eligible for the HPV vaccination, with no specific gender or age restrictions; (2) involved a gain-loss framing of intervention or combined with other message stimulation modalities; (3) compared gain framing with loss framing in groups, compared gain-loss framing against other message framing or against no message framing; (4) measured the effect of gain-loss framing on vaccination and the differences between them with attitudes, intentions, or behaviors as outcome evaluation modalities; (5) were intervention studies (including randomized controlled trials [RCTs] and quasiexperimental studies) published in English. Duplicate publications and publications with missing data were excluded.

We imported the literature into EndNote 21 to remove duplicates, then 2 researchers independently screened the documents and cross-checked them by reading the titles, abstracts, and full texts. Any disagreements were resolved by discussion or consultation with a third researcher.

### Data Extraction and Analysis

Data extraction was performed by 2 researchers, and any disagreements that arose were adjudicated by a third researcher. The following content was extracted: (1) basic information of the included studies, such as the first author, year of publication, country, subgroup, theoretical model, and methods or means of message dissemination; (2) baseline characteristics of the population, including the sample size, gender, and age; (3) outcome indicators and main findings. Due to the statistically significant heterogeneity of the publications, quantitative synthesis and a meta-analysis could not be accomplished; therefore, this study focused on the qualitative synthesis. Data extraction was performed using Microsoft Excel 2022.

### Quality Assessment

Two researchers independently evaluated the risk of bias for included studies and cross-checked the results, requesting a third researcher to negotiate a solution if they could not reach mutual agreement. The risk of bias was evaluated using the Cochrane Collaboration Risk of Bias (RoB) 2 [25]. For quasiexperimental studies, the selective bias item was rated as high risk because randomization was not conducted [26].

## Results

### Literature Search

**Figure 1:**
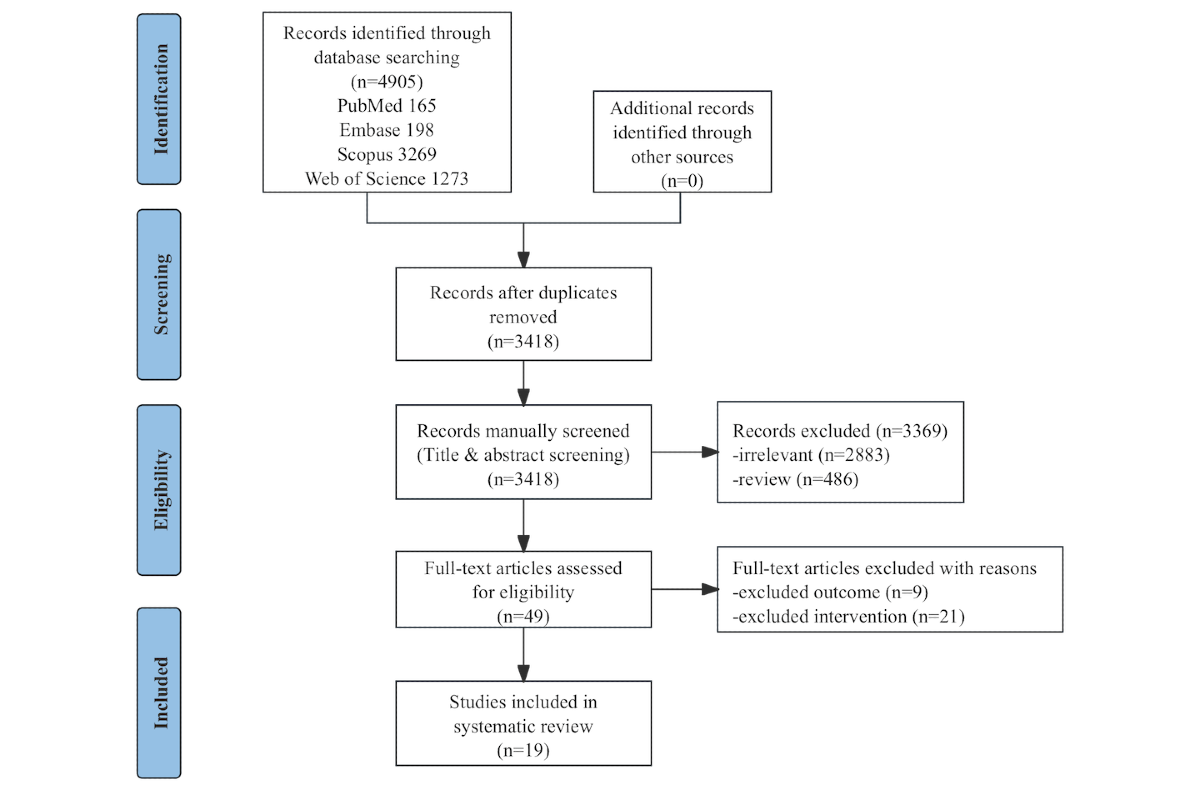
PRISMA (Preferred Reporting Items for Systematic Reviews and Meta-Analyses) flow diagram.

The preliminary screening obtained 4905 relevant studies. After duplicates were removed, the titles and abstracts of 3418 studies were screened, and the full text was screened for 49 of those studies. Finally, 19 studies were included [27-45], 2 of which were quasiexperimental studies that met the inclusion criteria [33,40]. The flowchart of the screening process is shown in Figure 1 [22].

### Characteristics of the Included Studies

The included studies were published between 2007 and 2023; 14 publications were from the United States [27-29,31,32,34-37,39,41-44], 2 were from China [38,45], and the other 3 were from Canada [33], Ireland [30], and Singapore [40]. The number of participants ranged from 72 to 739, with 5124 participants in total; the average age ranged from 18 years to 42 years; and 69.1% (3541/5124) of the participants were female.

Participants were categorized into gain-framing or loss-framing groups in 8 studies [27,30-32,35,37,40,44], and the remaining 11 publications added various message stimulation modalities or control groups [28,29,33,34,36,38,39,41-43,45]. Of the 19 studies, 9 delivered messages online in the form of videos or web pages, for example [32-35,39-42,45], and the rest implemented offline interventions via traditional printed materials like booklets and advertisements [27-31,36-38,43,44]. All the studies were based on prospect theory [27-45], and some of the studies applied the health belief model [28,34,36] or theory of planned behavior [30,34]. Participant outcomes were assessed in 13 studies [27-29,32,34-36,38-40,42,43,45], and 6 papers focused on participants’ attitudes, intentions, or behaviors toward their children's HPV vaccinations [30,31,33,37,41,44]. Intentions toward HPV vaccination were discussed in 13 studies [27-29,31,32,37-42,44,45], 5 studies described attitudes on HPV vaccination [30,33,35,36,43], and 1 study evaluated HPV vaccination behaviors directly [34]. Table 1 summarizes the basic information of the included literature.

**Table 1:** Characteristics of eligible studies about human papillomavirus (HPV) vaccination in the systematic review.

First author, year	Country	Sample, n (M^a^ or F^b^)	Sample age (years), mean (SD)	Vaccine recipient	Vaccine recipient age (years), mean (SD)	Group		Theory or model		Format (channel)		Results collection time	
Gerend, 2007 [27]	US	121 (F)	19.03 (1.09)	Self	—^c^		Gain or loss		PT^d^		Health booklet (offline)		Post intervention
Gerend, 2008 [28]	US	237 (F)	18.6 (1.1)	Self	—		Gain or loss and 1 shot or 6 shots		PT, HBM^e^		Booklet (offline)		Postintervention
Gerend, 2009 [29]	US	126 (M)	19.9 (1.9)	Self	—		Gain or loss and gray or red		PT		Binder (offline)		Pre- and postintervention
Fahy, 2010 [30]	Ireland	72 (F)	41.56 (5.81)	Daughter	8-16^f^		Gain or loss		PT, TPB^g^		Study booklet (offline)		Post intervention
Lechuga, 2011 [31]	US	150 (F)	33.72 (7.95)	Daughter	10.94 (4.01)		Gain or loss		PT		Laminated brochures (offline)		Pre- and postintervention
Nan, 2011 [32]	US	229 (M: 129; F: 100)	20.18 (1.47)	Self	—		Gain or loss		PT		Study website (online)		Postintervention
Gainforth, 2012 [33]	Canada	367 (M, F)	42.55 (4.73)	Daughter or son	11.32 (2.61)		Gain, loss, or mixed		PT, PMT^h^, HSM^i^		Messages (online)		Postintervention
Gerend, 2012 [34]	US	739 (F)	21 (1.8)	Self	—		Gain, loss, or no framed information		PT, HBM, TPB		Video (offline + online)		10 months postintervention
Nan, 2012 [35]	US	383 (M: 171, F: 212)	20.05 (1.45)	Self	—		Gain or loss		PT		Web page (online)		Postintervention
Park, 2012 [36]	US	108 (M: 27, F: 81)	20.5 (1.08)	Self	—		Gain or loss and high risk or low risk		PT, HBM		Vaccine advertising (offline)		Postintervention
Nan, 2016 [37]	US	193 (M: 52, F: 141)	36.2 (9.19)	Daughter or son	9-17^f^		Gain or loss		PT		Booklet (offline)		Postintervention
Wen, 2016 [38]	China	156 (M: 56, F: 100)	19.83 (0.91)	Self	—		Gain or loss and present or future		PT, CLT^j^, TDT^k^		Text-based message (offline)		Postintervention
Lee, 2017 [39]	US	142 (M: 30, F: 112)	22.44 (1.22)	Self	—		Gain or loss and SNSs^l^ or traditional media		PT, HBM		Web page (online)		Postintervention
Kim, 2018 [40]	Singapore	226 (F)	20.39 (1.57)	Self	—		Gain or loss		PT, RFT^m^		Web page (online)		Postintervention
Liu, 2018 [41]	US	431 (F)	30.16 (6.38)	Daughter or son	N/A^n^		Gain, loss, or narrative		PT		Newsletters (online)		Postintervention
Liu, 2018 [42]	US	455 (M, F)	20.42 (3.06)	Self	—		Gain or loss and English or Chinese		PT, CTR^o^, CCT^p^		Video (online)		Postintervention
Kim, 2020 [43]	US	347 (M: 132, F: 215)	22.2 (2.62)	Self	—		Gain or loss and future-thinking, past-thinking, or no-thinking		PT, EFM^q^, EFT^r^		Health message (offline)		Postintervention
Richards, 2021 [44]	US	184 (M: 50, F: 134)	36.13 (9.07)	Daughter or son	9-17^f^		Gain or loss		PT, PRT^s^		Pamphlet (offline)		Postintervention
Huang, 2023 [45]	China	458 (M)	26.36 (2.88)	Self	—		Gain or loss and self, other, or self-other		PT		Posters (online)		Postintervention

^a^M: male.

^b^F: female.

^c^Not applicable because the vaccine recipient sample is the same as the overall study sample.

^d^PT: prospect theory.

^e^HBM: health belief model.

^f^Range.

^g^TPB: theory of planned behavior.

^h^PMT: protection motivation theory.

^i^HSM: heuristic-systematic model.

^j^CLT: construal level theory.

^k^TDT: temporal discounting theory.

^l^SNSs: social networking sites.

^m^RFT: regulatory focus theory.

^n^N/A: not available.

^o^CTR: cultural theory of risk.

^p^CCT: cultural cognition thesis.

^q^EFM: emotions-as-frame model.

^r^EFT: episodic future thinking.

^s^PRT: psychological reactance theory.

### Quality Assessment of the Included Studies

**Figure 2:**
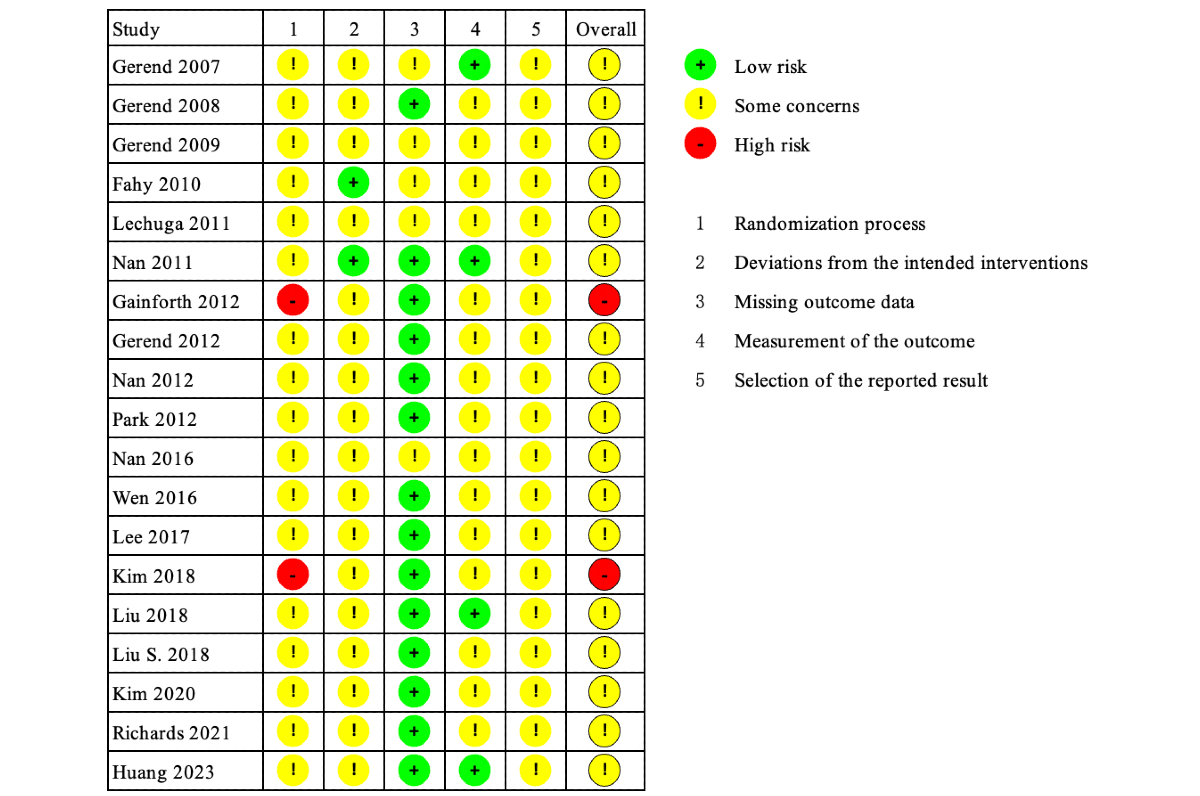
Risk of bias summary [27-45].

Among the 19 RCTs included, based on the RoB 2 assessment results, 2 studies were judged to have a high risk of bias, while the remaining 17 may have a risk of bias. Although 17 studies used randomization methods, none of them specified the implementation details for the randomization [27-29,31,32,34-39,41-45]. The random sampling methods in 2 studies were not clarified [33,40], while 2 studies reported specific allocation concealment measures [30,32]. Figure 2 summarizes the results of the risk of bias assessments for the included studies.

### Main Effects of Message Framing

Participant attitudes, intentions, or behaviors toward HPV vaccination were reported in the included literature. Of the 18 studies that evaluated attitudes or intentions toward vaccination, all of them assessed outcomes right after the intervention [27-33,35-45]. A single study measured physical behaviors for HPV vaccination 10 months postintervention [34]. The components of the intervention were related to the benefits of HPV vaccination and the losses of not getting the HPV vaccine. However, the diversity in the measuring instruments and statistical variation across the 19 studies resulted in significant heterogeneity, which prevented quantitative data synthesis. Table 2 summarizes the main findings of the included literature.

Of the 19 studies, 18 reported main or interaction effects of gain-loss framing on vaccination intentions [27-33,35-45]: 8 were focused on the impact of gain-loss framing–based interventions for HPV vaccination intentions [28,29,31,33,36,37,42,43], and 2 of the studies found a significant main effect of gain-loss framing on HPV vaccination intentions, which increased in both the gain-framing and loss-framing groups, as participants’ vaccination intentions rose from baseline [28,31]. The gain-loss framing was shown to have no major effect on vaccination intentions in 5 studies [29,33,36,42,43], and mothers' intentions to have their daughters vaccinated were not affected by the gain-loss framing [30]. The differences between gain-framing and loss-framing on HPV vaccination intentions were compared in 12 trials [27-29,31,32,35-39,41,44], of which 8 studies found that the loss framing produced higher vaccination intentions than the gain framing [27-29,32,35-37,39], 3 studies showed no statistically significant difference between the 2 groups after comparison [31,38,41], and 1 RCT revealed loss framing significantly reduces the intention to vaccinate [44].

The influence of message framing on attitudes toward HPV vaccination were explored by 5 studies. [30,33,35,36,43]. Gain-loss framing had no significant impact on attitudes toward HPV vaccination directly [30,33,43]. In contrast, Park [36] found that gain-loss framing had a major effect on HPV vaccination attitudes. Furthermore, a comparison of the effect of 2 message frames on vaccination attitudes revealed that loss framing produced better attitudes about vaccination than gain framing [35].

In 1 study that measured HPV vaccination rates, 6% of participants received one or more doses of an HPV vaccine 10 months after the intervention, and the comparison of the difference in vaccination rates between message frames showed that the rates of HPV vaccination were almost the same with the different message frames [34].

**Table 2:** Main findings of outcome variables and relevant moderator or mediator variables.

First author, year	Outcome variables	Moderator or mediator variables	Main findings
Gerend, 2007 [27]	Intention: a 6-point scale with 5 items	Sexual behavior, motivational orientation	The loss-framed message leads to higher vaccination intentions than the gain-framed message for women engaging in risky sexual behaviors or with high avoidance motivation.
Gerend, 2008 [28]	Intention: a 6-point scale with 5 items	Behavioral frequency (1 shot or 6 shots); perceived severity, benefits, barriers, susceptibility, and self-efficacy	Message framing has a significant main effect on HPV^a^ vaccination intentions. Participants exposed to the loss-framed message had higher vaccination intentions than those exposed to the gain-framed message. Message framing interacted with behavioral frequency, and the loss-framed message led to greater vaccination intentions than gain-framed message in the case of 1 vaccine shot. Perceived barriers and severity have no mediating effect; perceived susceptibility and self-efficacy can predict vaccination intentions, while perceived benefits cannot.
Gerend, 2009 [29]	Intention: a 6-point scale with 5 items	Color priming (gray or red)	Message framing does not have a major impact on vaccination intentions; of the framed messages labeled with red rectangular boxes, those who read the loss-framed message had higher vaccination intentions than those who read the gain-framed message.
Fahy, 2010 [30]	Intention: a 7-point scale with 3 items; attitude: 3 bipolar semantic differential scales	Attitude, normative beliefs;, PBC^b^	No main effect of message framing on vaccination attitudes. Mothers’ high intentions to have their daughters vaccinated is not influenced by message framing effects. Attitude, normative beliefs, and PBC explain 69.5% of the variation in intentions.
Lechuga, 2011 [31]	Intention: a 7-point scale with 5 items	Ethnic group: Hispanic, non- Hispanic White, African American	Vaccination intention is higher with gain framing or loss framing than at baseline. For the African Americans and Hispanics, the loss-framed message generates higher vaccination intentions; for non-Hispanic Whites, there was no difference between the 2 frames.
Nan, 2011 [32]	Intention: a 7-point scale with 3 items	Motivational orientation	The loss-framed message leads to higher vaccination intentions than the gain-framed message in avoidance-oriented participants.
Gainforth, 2012 [33]	Attitude: a 7-point scale with 5 semantic differential items; intention: a 7-point scale including 6 items	Sex of the parent and child	Parents' intentions to vaccinate their children against HPV is not significant. There was no effect based on gender, message frame, and parents’ attitudes.
Gerend, 2012 [34]	Behavior: HPV vaccination rates	Perceived susceptibility, severity, benefits; safety concerns; cost; attitudes; norms; self-efficacy	HPV vaccination rates are almost the same across different message frames. Perceived susceptibility, perceived safety, and vaccine cost can predict vaccine uptake, while perceived severity and benefits cannot.
Nan, 2012 [35]	Attitude: a 7-point scale with 3 semantic differential items; intention: a 7-point scale with 3 items	Time orientation	The loss-framed message leads to greater vaccination attitudes and intentions than the gain-framed message. Participants with a future-mind had more favorable intentions and attitudes to be vaccinated. For participants with a present-mind, a loss-framed message leads to greater vaccination attitudes than a gain-framed message, while the 2 frames perform equally in future-minded participants.
Park, 2012 [36]	Attitude: a 7-point scale with 9 semantic differential items; intention: a 7-point scale with 4 items	Perceived risk	Message framing has a main effect on HPV vaccination attitudes. Participants exposed to the loss-framed message had higher vaccination intentions. Participants with high risk perceptions have a strong intention to be vaccinated.
Nan, 2016 [37]	Intention: 3 scoring items	Perceived susceptibility	Message framing cannot predict vaccination intentions. The loss-framed message produced more vaccination intentions than the gain-framed message when perceived susceptibility was low, whereas the gain-framed message produced more vaccination intentions than the loss-framed message when perceived susceptibility was high.
Wen, 2016 [38]	Intention: a scale with 4 items	Temporal distance; prior knowledge	The interaction effects between message framing and temporal distance were not significant. There is no difference between loss-framed messages, gain-framed messages, future-framed messages, and present-framed messages in terms of behavioral intention. Prior knowledge has main effects on behavioral intention.
Lee, 2017 [39]	Intention: a 7-point scale with 4 items	Media channel	The loss-framed message produced more vaccination intentions than the gain-framed message. Participants showed a higher level of behavioral intention to get HPV immunization after viewing the loss-framed message post on SNSs^c^.
Kim, 2018 [40]	Intention: a 5-point scale with 3 items	Transportation, self-referent, emotions, free vaccine, paid vaccine	There was an indirect impact of message framing on vaccination intentions through transportation (free vaccine). Self-referent emotions mediate message framing and vaccination intentions.
Liu, 2018 [41]	Intention: a 7-point scale with 3 items	Time orientation	The loss-framed message produces high vaccination intention, but the difference is not significant. The interaction effect between loss-framed messages and time orientation cannot predict vaccination intention.
Liu, 2018 [42]	Intention: a 7-point scale with 3 items	Cultural worldview (English or Chinese)	Message framing does not have a major impact on vaccination intentions. An individualistic worldview was positively associated with Chinese participants’ willingness to be vaccinated and negatively associated with US participants' willingness to be vaccinated.
Kim, 2020 [43]	Intention: a 7-point scale with 3 items; attitude: a 7-point scale with 5 semantic differential items	Anticipated regret, EFT^d^ (future-thinking, past-thinking, or no-thinking)	The direct effect of message framing on attitudes and intentions toward HPV vaccination is not significant. Anticipated regret has an indirect effect in message framing and HPV vaccination attitudes and intentions. EFT and message framing interact on attitudes toward HPV vaccination. Future thinking produces more favorable attitudes than past thinking in the gain-framed message.
Richards, 2021 [44]	Intention: a 5-point scale with 3 items	Perceived efficacy	For parents with low perceived efficacy, the loss frame (compared with the gain frame) significantly reduced the intention to vaccinate.
Huang, 2023 [45]	Intention: a 7-point scale with 7 items	Reference point (self, other, or self-other)	Message framing has no moderating effect between reference points and behavioral intentions.

^a^HPV: human papillomavirus.

^b^PBC: perceived behavioral control.

^c^SNSs: social networking sites.

^d^EFT: episodic future thinking.

### Moderator or Mediator Variables of Message Framing

Each of the included studies explored moderating or mediating factors that affect the efficacy of message framing. A study by Fahy and Desmond [30] observed the mediating role between the theory of planned behavior and message framing and discovered that attitudes, normative beliefs, and perceived behavioral control explained 69.5% of the variation in vaccination intentions. The mediating effects of personal perceptions between message framing and intentions or behaviors were examined by 5 studies [28,34,36,37,44]. Gerend et al [28] demonstrated that there was no mediating effect between perceived barriers or perceived severity and message framing; although perceived susceptibility and self-efficacy were able to predict vaccination intentions, perceived benefit did not. Perceived susceptibility, perceived safety, and vaccine cost can predict vaccination rates, whereas perceived severity and benefit do not [34]. Participants with a high risk perception had a strong intention to be vaccinated [36]. Nan et al [37] discovered that loss framing produced more vaccination intentions than gain framing when perceived susceptibility was low, while when perceived susceptibility was high, gain framing produced more vaccination intentions than loss framing. For parents with low perceived efficacy, loss framing significantly reduced intentions to vaccinate [44]. Kim et al [40] reported that message framing had an indirect effect on vaccination intentions mediated by transportation, self-inferred emotions, and anticipated regret. In addition, there was no impact of gender on vaccination attitudes [33], and prior knowledge had a major effect on behavioral intentions [38].

Nan [35] and Gerend et al [28] discovered that loss framing was associated with higher intentions to vaccinate than gain framing among participants with high avoidance motivation, which also applied to women engaging in risky sexual behaviors [27]. Nan [35] investigated the interaction between message framing and temporal orientation and found a marked interaction between them. The 2 frames worked equivalently in future-minded participants, and participants with a future-minded perspective possessed more intentions and attitudes toward vaccination. For present-minded participants, loss framing resulted in more vaccination attitudes than gain framing. By contrast, another study identified an interaction between loss framing and temporal orientation, which did not predict intentions to vaccinate [41]. Lechuga et al [31] compared the effects of message framing on different racial populations and found that, for African Americans and Hispanics, loss framing produced higher vaccination intentions, whereas for non-Hispanic Whites, there was no difference between the 2 types of message framing. Liu et al [42] identified no remarkable interaction between cultural worldviews and message framing; however, after categorizing by population, a positive interaction was found between individualistic worldviews and vaccination intentions of Chinese recipients and a negative interaction between individualistic worldviews and vaccination intentions of American recipients.

The interaction between message framing and behavioral frequency was explored by Gerend et al [28]. They found that loss framing contributed to higher vaccination intentions than gain framing in cases of 1-shot vaccination; however, there was no difference between the 2 message frames for the 6 doses required. Another study used colored primers to test the effect of message frames labeled red or gray on participants’ HPV vaccination intentions. The finding that people who read loss-framed messages with red rectangular labels had higher vaccination intentions than those who read gain-framed messages (with noncolored labels) suggests that the combination of loss framing and a red label may have had a stronger influence on vaccination intentions than gain framing alone [29]. After conducting an intervention with different transmission media, Lee and Cho [39] found that participants expressed higher levels of behavioral intention for HPV vaccination when viewing loss-framed messages posted on social networking media. Kim et al [40] found an interaction between episodic future thinking and message framing, with future-thinking messages producing more favorable attitudes than past-thinking messages in gain-framed messages and no differences between future and past messages in the loss-framed situation on vaccination attitudes. In contrast, Wen and Shen [38] discovered that message framing and temporal distance interacted insignificantly, and there were no significant differences between loss-framed, gain-framed, future-framed, and present-framed messages on vaccination intentions. Huang and Li [45] added information specific to different vaccination targets in message frames and showed that no moderating effect was found between different reference points of message frames and vaccination intentions.

## Discussion

### Principal Findings

The construction of message framing can effectively affect the public’s health attitudes and increase their intentions to practice healthy activities [46]. Therefore, we conducted a systematic review of 19 studies on the persuasive effects of message framing on HPV vaccination and summarized the effects of framing-based message stimulation approaches on participants’ attitudes, intentions, or behaviors toward HPV vaccination. Gain-loss framing had no clear effect on vaccination attitudes or intentions. Loss framing showed a weak advantage in improving HPV vaccination attitudes or intentions, but the evidence was not strong enough to draw definitive conclusions. The gain-loss framing could combine various message stimulus contents or modalities to affect HPV vaccination attitudes, intentions, or behaviors distinctively. The impact of gain-loss framing on HPV vaccination behaviors could not be determined due to the limited number of studies and the qualitative nature of the analysis.

Previous reviews have established the advantage of loss framing for improving vaccination attitudes and intentions [47]. However, they did not specifically address the unique context of HPV vaccination. Additionally, we included recent studies published after the last major meta-analysis, ensuring that our review reflects the most up-to-date evidence. Although the direct effect of framing on attitudes and intentions could not be quantitatively assessed due to the limitations of our analysis, our review provides nuanced insights and highlights potential gaps in the existing knowledge. By synthesizing the available qualitative data, we contribute to a more complete understanding of the role of framing in vaccination promotion.

Our study showed that loss framing was more effective than gain framing at improving HPV vaccination attitudes and intentions. However, the distinction in persuasion effects between gain framing and loss framing is not as simple as we assumed. Earlier research on gain-loss framing did not uniformly and clearly present which one was more effective. Rothman and Salovey [48] conducted a literature review of a large number of studies, combing the content of previous studies, and proposed that the effectiveness of message framing must be focused on the specific scenarios of the study design. The direction and strength that specific framing imposes on intentions to adopt healthy behaviors may have diverse or conflicting results across studies depending on the scene setting, researcher manipulation, measurements, and individual traits like educational level and age of the participants. Some studies have suggested no significant difference between gain framing, which emphasizes the benefits of vaccination, and loss framing, which focuses on the potential risks of not vaccinating, in terms of motivating attitudes or intentions to vaccinate [31,38,41], and others argued that loss framing was more persuasive [27-29,32,35-37,39]. The conflicting findings explained part of our results, although the studies supporting the dominance of loss framing represented more than one-half of the literature, with 11 of the 19 included publications (57.9%) supporting this finding. However, any statistical differences remain uncertain due to the absence of quantitative data integration.

In this systematic review, there was no direct effect of gain-loss framing on vaccination attitudes nor intentions, which supported the results of previous studies [14,49]. All literature included in this systematic review explored moderating or mediating variables between gain-loss framing and vaccination attitudes, intentions, or behaviors. With loss framing, messages combining individualistic worldviews, vaccination frequency, and transmission via social media or labeling message stimulus elements in red could enhance vaccination intentions of participants [28,29,39,42]. Meanwhile, loss framing messages produced higher vaccination intentions for high avoidance motivators, women engaged in risky sexual behaviors, African Americans and Hispanics, present-minded subjects, and low perceived susceptibility participants [27,31,32,35,37]. In addition, attitudes, normative beliefs, perceived behavioral control, perceived susceptibility, self-efficacy, perceived safety, perceived high risk, transportation, self-inferred emotions, and anticipated regret mediated vaccination intentions with gain-loss framing. Earlier opinions have pointed out that a single framing to achieve the expected persuasive effect or to explain altered attitudes and behavioral intentions is not convincing enough. In reality, the architecture of segment information is complex and multifaceted, comprising various message frames that embody diverse design ideas. Conducting research on multiple message frames, therefore, represents a crucial direction for the advancement of message framing theory and practice [50]. Additional high-quality RCTs are needed to verify the accuracy of this result due to the amount of literature that discusses the same variables.

Notably, only 1 study discussed the impact of gain-loss framing on vaccination behavior. Therefore, we were not able to draw conclusions related to the effects of gain-loss framing on vaccination behavior, which provides inspiration for future investigations. Attitudes or intentions are predictors of behavior, and the examination of them is clinically relevant; nevertheless, the ultimate purpose of structuring messages is to instruct the recipients to behave in a particular way [51]. We ought to go beyond the predictors; changing vaccination behaviors and increasing HPV vaccination rates are the objective of implementing interventions.

### Limitations

There are several limitations in this systematic review. First, the absence of ongoing studies and only including documents published in English could not guarantee the inclusion of all eligible literature for the systematic review. Second, RoB 2 was selected as the risk of bias evaluation tool, which was influenced by major subjective factors. In addition, assessment tools for outcome indicators and statistical methods varied among the included literature, with a high degree of heterogeneity that diminishes the accuracy of final conclusions. Only 1 study measured behavioral change postintervention, and the other 18 studies assessed outcomes immediately after treatment, without sufficient time for individuals to assimilate and deliberate messages. In addition, the study provided an overview of the influence of attitudes, intentions, or behaviors toward HPV vaccination, but it was unable to clarify the specific causal relations of the 3 dependent variables. Instead, it had to follow former studies that regarded vaccination intentions as the strongest psychological motivation for behavioral occurrences. Due to these limitations, the results should be treated carefully.

### Conclusion

This systematic review suggests that loss-framing messages show promise for boosting vaccination attitudes and intentions more effectively than gain-loss framing. The latter did not consistently demonstrate statistically significant advantages. The influence of gain-loss framing on vaccination perspectives varies significantly among diverse ethnic populations, underscoring the importance of cultural considerations in messaging strategies. Integrating gain-loss framing with alternative communication approaches or delivery platforms produces a spectrum of outcomes on vaccine attitudes, intentions, and behaviors, highlighting the potential for tailored interventions. To enhance the efficacy of message-based interventions for vaccination promotion, a broader perspective is imperative. This includes targeting audiences across all age groups, educational backgrounds, socioeconomic strata, and the digital divide while also considering individual media preferences and online health literacy levels. Longitudinal studies are necessary to ascertain the sustained impact of message framing on vaccination behaviors, thereby enriching our understanding of framing effects over time. Future research should prioritize behavioral interventions accompanied by objective outcome measurements, fortifying the evidence base for the strategic application of message framing in public health campaigns.

## Appendix

Multimedia Appendix 1 PRISMA (Preferred Reporting Items for Systematic Reviews and Meta-Analyses) checklist.

## Abbreviations

HPV: human papillomavirus
PRISMA: Preferred Reporting Items for Systematic Reviews and Meta-Analyses
RCT: randomized controlled trial
RoB: risk of bias
