# Correction: Effects of Message Framing on Human Papillomavirus Vaccination: Systematic Review

**DOI:** 10.2196/68783

**Published:** 2024-11-15

**Authors:** Jie Gong, Dandan Gu, Suyun Dong, Wangqin Shen, Haiou Yan, Juan Xie

**Affiliations:** 1 Department of Nursing Affiliated Hospital of Nantong University Nursing and Rehabilitation School of Nantong University Nantong China; 2 School of Nursing and Rehabilitation Nantong University Nantong China

In “Effects of Message Framing on Human Papillomavirus Vaccination: Systematic Review” (J Med Internet Res 2024;26:e52738) one error was noted.

In the Methods section of the text, the PROSPERO registration number was revised. Therefore, the following sentence:

The study protocol was registered in PROSPERO (number CRD42063451612).

Has been revised to:

The study protocol was registered in PROSPERO (number CRD42023451612).

The correction will appear in the online version of the paper on the JMIR Publications website on November 15, 2024, together with the publication of this correction notice. Because this was made after submission to PubMed, PubMed Central, and other full-text repositories, the corrected article has also been resubmitted to those repositories.

